# Incidence and Risk Factors of Recurrent *Clostridioides difficile* Infection in Patients With Cirrhosis

**DOI:** 10.14309/ctg.0000000000000189

**Published:** 2020-07-13

**Authors:** Parkpoom Phatharacharukul, Russell D. Purpura, Devika Gandhi, Huiping Xu, Katie Bickett-Burkhart, Naga Chalasani, Monika Fischer, Eric S. Orman

**Affiliations:** 1 Division of Gastroenterology and Hepatology, Indiana University School of Medicine, Indianapolis, Indiana, USA;; 2 Department of Medicine, Indiana University School of Medicine, Indianapolis, Indiana, USA;; 3 Department of Biostatistics, Indiana University School of Medicine, Indianapolis, Indiana, USA;; 4 Regenstrief Institute, Inc, Indianapolis, Indiana, USA.

## Abstract

**INTRODUCTION:**

*Clostridioides difficile* infection (CDI) is common in patients with cirrhosis and is associated with poor outcomes. CDI risk factors in this population have been well characterized; however, risk factors of recurrent CDI (R-CDI) after treatment have not been explored. We sought to estimate the incidence of R-CDI and its associated risk factors in patients with cirrhosis.

**METHODS:**

We performed a cohort study of patients with cirrhosis hospitalized with CDI between 2012 and 2016. We collected patient characteristics, including detailed information on the CDI, features of the underlying liver disease, and outcomes including R-CDI, hospital readmission, and mortality. R-CDI was defined as CDI occurring 2–8 weeks after the initial episode. Cox proportional hazards model was used to identify variables independently associated with the outcomes.

**RESULTS:**

A total of 257 hospitalized patients with cirrhosis and CDI were included. CDI was community associated in 22.6%. The incidence of R-CDI was 11.9%. R-CDI was not significantly associated with medications at hospital admission or discharge. Independent risk factors of R-CDI included increased Charlson Comorbidity Index (hazard ratio [HR] 1.30; 95% confidence interval [CI]: 1.09–1.55) and use of lactulose (HR 2.58; 95% CI: 1.09–6.09). The 30-day readmission rate was 37%, and readmission was associated with increased Charlson Comorbidity Index (HR 1.12; 95% CI: 1.03–1.23) and Model for End-Stage Liver Disease score (HR 1.04; 95% CI: 1.01–1.07). The 90-day mortality was 22.8%.

**DISCUSSION:**

In patients with cirrhosis, R-CDI is associated with comorbidity burden and lactulose use. Attention to these factors might aid clinicians in efforts to prevent R-CDI and improve outcomes in this population.

## INTRODUCTION

*Clostridioides difficile* infection (CDI) is a common diarrheal pathogen with increasing incidence and severity in both outpatient and inpatient settings (1,2). Despite significant advances in CDI detection and treatment, the resultant healthcare costs continue to rise, and outcomes continue to worsen (3,4). Common CDI risk factors include hospitalization, immunosuppression, advanced comorbidities, and the use of medications such as antibiotics and proton pump inhibitors (5). These risk factors are highly prevalent in patients with liver cirrhosis, who are particularly vulnerable to CDI. Patients with cirrhosis have high rates of hospitalization and are often exposed to antibiotics for prophylaxis and treatment of frequent infections (6,7).

Traditional first-line therapeutic agents for CDI include metronidazole and oral vancomycin, with response rates ranging from 65% to 98% depending on disease severity (8,9). However, recent updates to clinical practice guidelines no longer endorse metronidazole and, instead, suggest either oral vancomycin or fidaxomicin as first-line therapy for both severe and nonsevere cases. This change was based on improved symptom response and mortality with vancomycin when compared with metronidazole (10,11). Treatment failure remains a major concern; recurrent CDI (R-CDI) results in increased hospital length of stay, readmissions, and costs (12). These poor outcomes are magnified in patients with cirrhosis. For the general population hospitalized with CDI, average length of stay is 13 days, inpatient mortality is 8%, and 30-day readmissions occur in 20%; in patients with cirrhosis, these figures are significantly higher: at 14 days, 14%, and 35%, respectively (8,12,13). In patients with cirrhosis, CDI is also an independent risk factor of mortality, similar to other cirrhosis complications such as hepatic encephalopathy, variceal hemorrhage, and spontaneous bacterial peritonitis. Despite this growing evidence for identifying risk factors of CDI and outcomes in cirrhosis, there remains a gap in the literature exploring risk factors of R-CDI in this population.

We, therefore, sought to identify risk factors of R-CDI and its associated outcomes in patients with cirrhosis and CDI. To achieve this goal, we performed a cohort study of hospitalized patients with cirrhosis and CDI between 2012 and 2016, examining clinical characteristics and outcomes during and after hospitalization.

## METHODS

### Study design and patients

The study protocol was approved by the Indiana University Institutional Review Board. We performed a retrospective cohort study of adult patients (aged ≥18 years) admitted to Indiana University Hospital between January 1, 2012, and December 31, 2016, with a diagnosis of cirrhosis and CDI. Indiana University Hospital is a tertiary referral center and the only liver transplant program in the state. Patients were followed up for 90 days from the time of the CDI diagnosis to ascertain outcomes. The electronic medical record was screened for hospitalized patients with both diagnoses using diagnostic codes for each condition and positive laboratory results for CDI (a rapid membrane enzyme immunoassay for the simultaneous detection of *C. difficile* glutamate dehydrogenase antigen and toxins A and B in a single reaction). Patients identified in this way were then manually reviewed to confirm the diagnoses. We also required patients to have compatible symptoms (i.e., patients with a positive laboratory result without diarrhea or other clinical features of CDI were not included). To prevent false positive results at our hospital, laboratory policy mandates that all laxatives (including lactulose) must be stopped at least 48 hours before CDI testing. Cirrhosis was confirmed by liver histology or on the basis of compatible clinical, laboratory, and imaging findings. CDI was confirmed on the basis of compatible symptoms and a positive stool toxin enzyme immunoassay or polymerase chain reaction. We excluded patients with previous CDI, those on treatment for CDI before admission, those hospitalized <48 hours, those with inflammatory bowel disease, and those with previous liver transplants.

### Outcomes

The primary study outcome was R-CDI, defined as CDI occurring within 14–56 days of the initial CDI diagnosis date (14). As in the inclusion criteria for initial CDI, R-CDI was defined based on compatible symptoms accompanied by a positive laboratory test. Secondary outcomes included mortality within 90 days of CDI diagnosis and readmissions within 30 days of hospital discharge.

### Variables

We collected multiple variables that could be associated with patient outcomes. These variables included demographic information (age, sex, and race) and medical history (body mass index, liver disease etiology, Charlson Comorbidity Index (15), active alcohol use, presence of end-stage renal disease, and presence of concurrent infections (16)). We also collected measures of liver disease severity on admission (Child–Pugh score (17), Model for End-Stage Liver Disease [MELD] score (18)) and cirrhosis complications (previous transjugular intrahepatic shunt, hepatocellular carcinoma, and spontaneous bacterial peritonitis). Medications of interest on admission and at discharge included proton pump inhibitors, histamine 2 receptor antagonists, antibiotics, nonselective β-blockers, lactulose, probiotics, and polyethylene glycol 3350. Index hospitalization data included the primary reason for admission, admission to intensive care, length of stay, and discharge disposition. Characteristics of the initial CDI included the type of CDI (community-associated; community-onset, healthcare facility-associated; and healthcare facility-onset CDI) (14), presence of severe or fulminant CDI, and CDI treatment. Severe and fulminant CDI were defined based on standard guideline definitions (14). For those with R-CDI, characteristics of the recurrence were also captured.

### Statistical analysis

Categorical variables were described using counts and percentages; continuous variables were described with means and SDs for normally distributed variables and with medians and interquartile ranges for nonnormally distributed variables. Because R-CDI is defined as occurring within 2–8 weeks of the initial CDI, for the primary R-CDI analysis, we excluded those who died within 2 weeks of the initial CDI and those without follow-up data beyond the initial 2 weeks. For the readmission analysis, we excluded those without follow-up data after discharge. Days to the outcome occurrence were examined using the Kaplan–Meier curve. The univariable associations between patient characteristics and outcome were evaluated using the Cox proportional hazards model. Firth bias correction method was used to address the monotone likelihood issue where parameter estimates converges to infinite because of small sample size and sparse data (19). Multivariable analysis was performed in which all patient characteristics with a univariable *P* < 0.25 were subjected to a forward stepwise variable selection process. A 2-sided *P* < 0.05 was considered significant. All statistical analyses were performed using SAS version 9.4 (The SAS institute, Cary, NC).

## RESULTS

### Baseline characteristics

**Figure 1.**
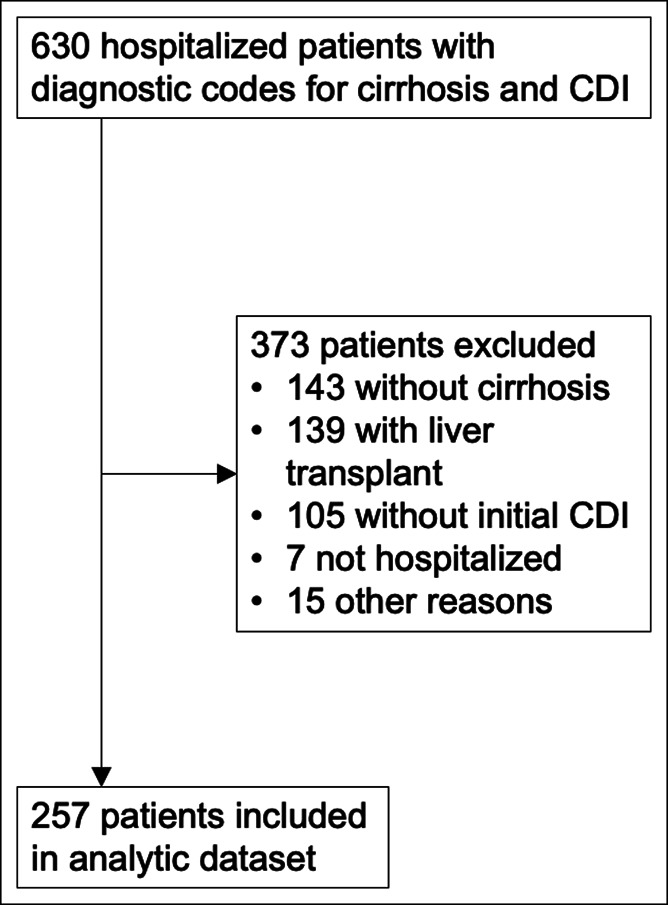
Flow diagram. The sum of the exclusion criteria is greater than the total number of patients excluded because some patients fulfilled multiple exclusion criteria. CDI, *Clostridioides difficile* infection.

Of 630 patients identified as having a diagnosis of cirrhosis and a hospitalization with CDI during the study period, 373 were excluded, leaving 257 patients for analysis (Figure 1). Patient characteristics are summarized in Table 1. The mean age was 58.5 years (SD 12.3), and 52.9% were men; 91.8% had Child-Pugh B or C cirrhosis, and the median MELD was 18 (interquartile range 12–24). The most common admission medications were proton pump inhibitors (55.6%), nonselective β-blockers (33.1%), lactulose (35.4%), and rifaximin (24.9%). Approximately 15% were taking fluoroquinolones. CDI was classified as healthcare facility-onset CDI in 40.9%; community-onset, healthcare facility-associated CDI in 29.6%; and community-associated CDI in 22.6%. CDI was severe in 62.6%; 51.4% received vancomycin, and 48.6% received metronidazole only. One patient received fidaxomicin, and 1 received fecal microbiota transplant.

**Table 1.**
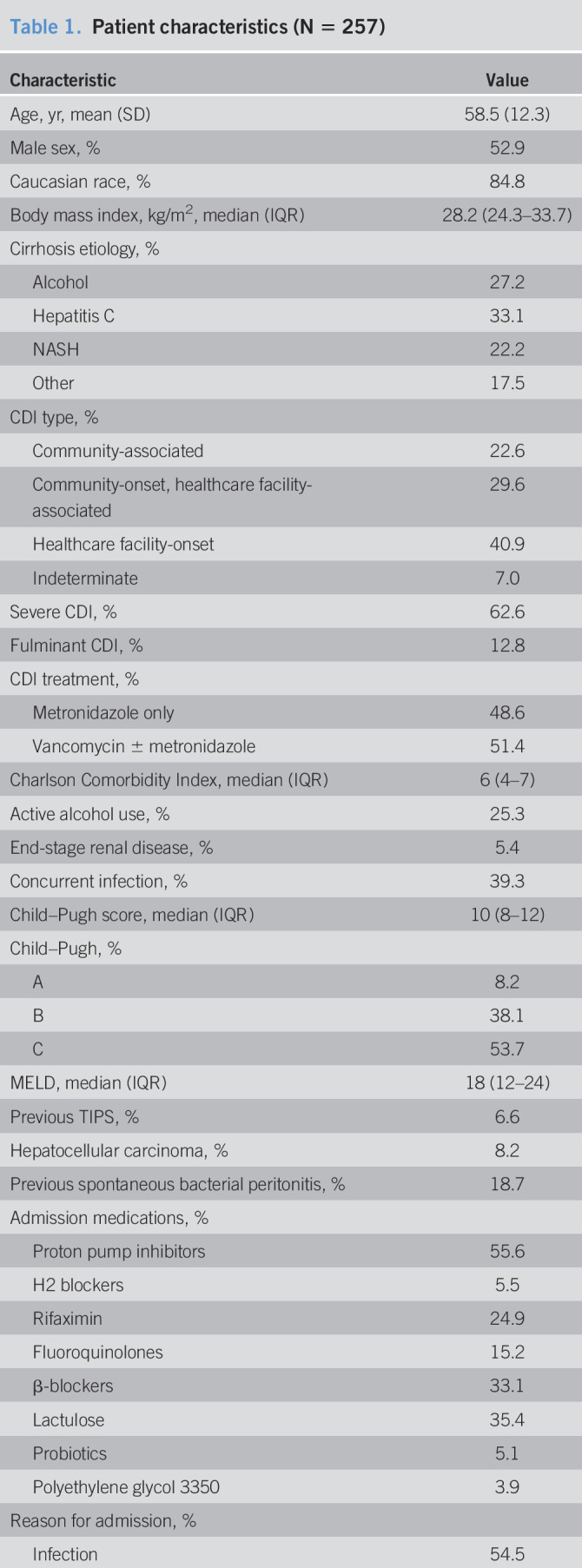

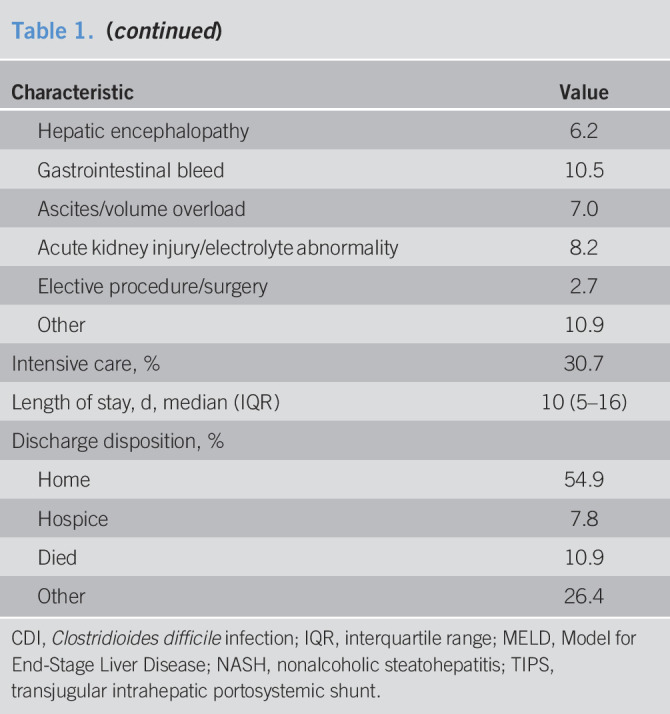
Patient characteristics (N = 257)

### Recurrent CDI

**Figure 2.**
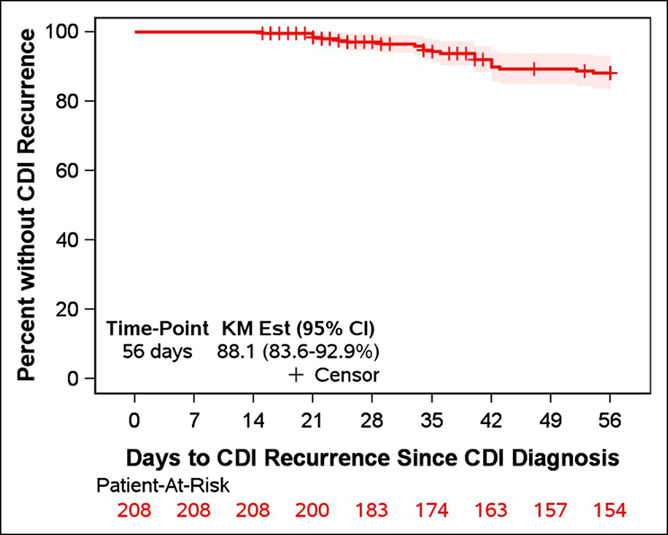
Kaplan–Meier plot of time to CDI recurrence. Patients were followed up from the time of initial CDI diagnosis. CDI, *Clostridioides difficile* infection.

Of the 257 patients, 28 died within 2 weeks, and 21 did not have follow-up data beyond 2 weeks, leaving 208 for analysis of R-CDI. A total of 22 patients developed R-CDI, resulting in an estimated R-CDI incidence of 11.9% (Figure 2). Univariable associations between admission factors and R-CDI are tabulated in Table 2. R-CDI was associated with increased age, nonhepatitis C etiology of cirrhosis, increased Charlson Comorbidity Index, increased length of hospital stay, and discharge to “other” location. There were no significant associations between R-CDI and medications at hospital admission or discharge. At discharge, 62% of patients were taking a proton pump inhibitor, with 14.5% R-CDI (compared with 7.6% in those not taking a proton pump inhibitor; *P* = 0.15). In addition, 12% were taking a fluoroquinolone at discharge, with 21.8% R-CDI (compared with 10.7% in those not taking a fluoroquinolone; *P* = 0.18). CDI recurred in the patient who received fidaxomicin and in the patient who received fecal microbiota transplant. In a multivariable model, stepwise variable selection identified 2 variables independently associated with R-CDI. A greater risk of R-CDI was associated with the use of lactulose (hazard ratio [HR] 2.58; 95% confidence interval [CI]: 1.09–6.09) and increased Charlson Comorbidity Index, where each point increase in the comorbidity index was associated with 30% greater hazard of R-CDI (HR 1.30; 95% CI: 1.09–1.55).

**Table 2.**
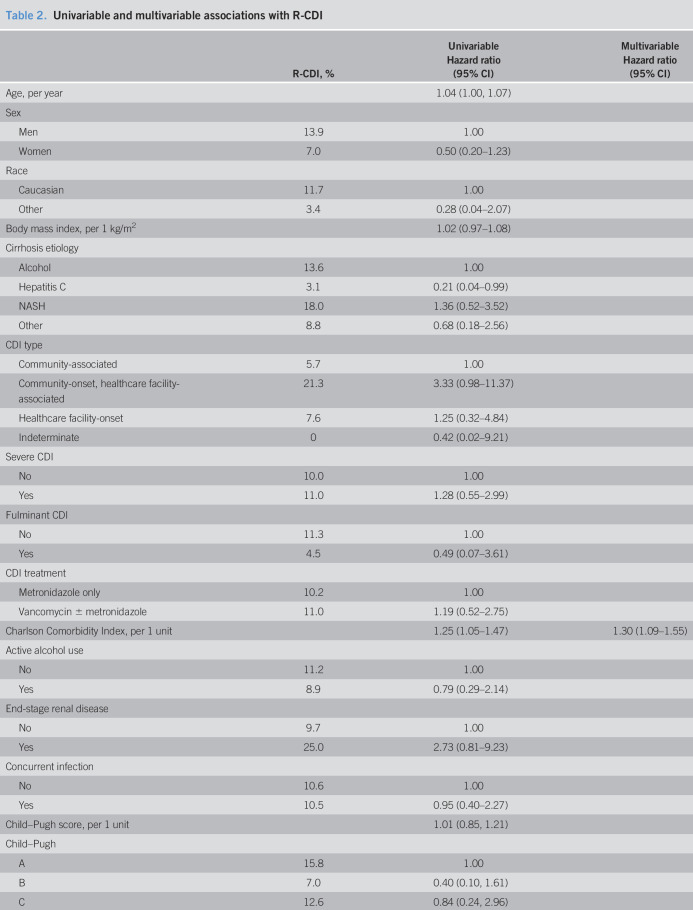

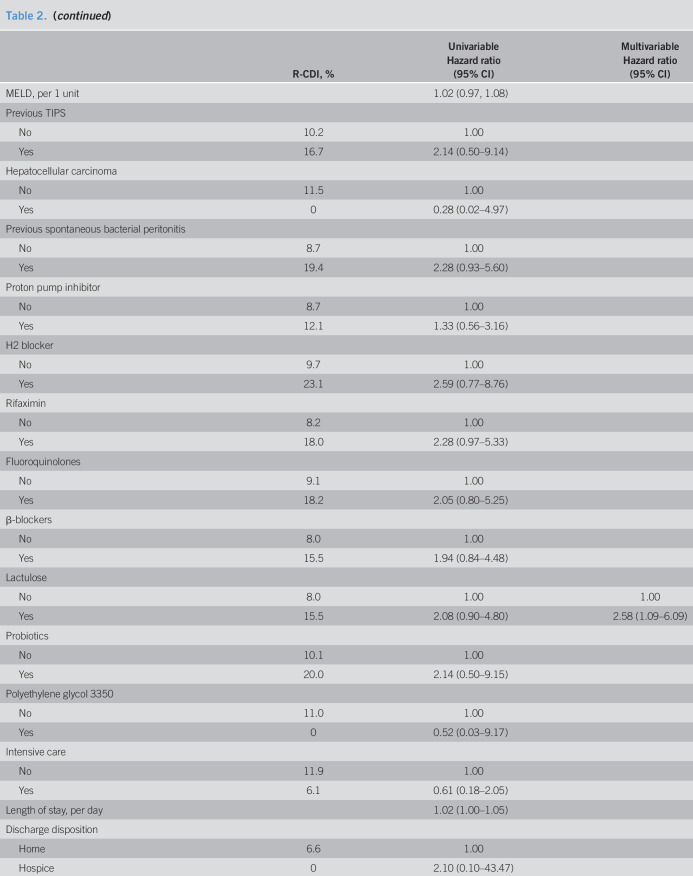

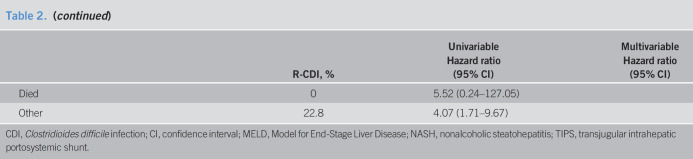
Univariable and multivariable associations with R-CDI

Among the 22 patients who developed R-CDI, 9 (40.9%) met criteria for severe CDI, and 1 patient developed fulminant colitis but was felt to be too ill for surgery and, thus, did not undergo colectomy; this patient died during the admission. Five patients (22.7%) required intensive care. For the recurrence, 4 patients received oral metronidazole only, 11 received oral vancomycin only, 3 received intravenous metronidazole only, 1 received oral fidaxomicin, and the remaining 3 received combination therapy. None received fecal microbiota transplant. Five patients (22.7%) died or enrolled in hospice care. Twelve of the 22 patients had multiple R-CDI ranging from 2 to 7 episodes.

### Thirty-day readmissions

**Figure 3.**
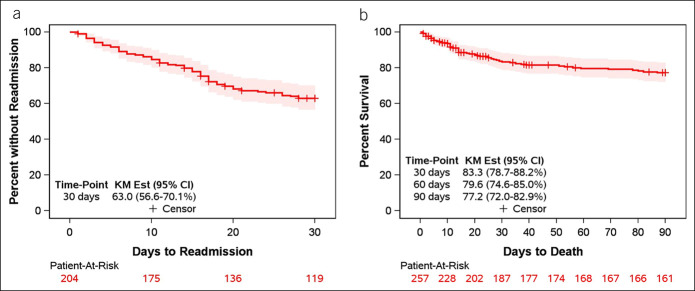
(**a**) Kaplan–Meier plot of time to hospital readmission. Patients were followed up from the time of index hospital discharge. (**b**) Kaplan–Meier plot of time to death. Patients were followed up from the time of initial *Clostridioides difficile* infection diagnosis.

Patients who died during the index admission or had no follow-up data after hospital discharge were excluded from the readmission analysis (n = 53), leaving 204 patients for analysis. Of these, 74 were readmitted to the hospital within 30 days, resulting in an estimated 37% readmission rate (Figure 3a). Readmissions were specifically for CDI in 12 patients, resulting in an estimated 6.2% readmission rate. Patients without R-CDI had a 30-day readmission rate of 32%. In univariable analysis (Table 3), readmissions were associated with presence of severe CDI, increased Charlson Comorbidity Index, increased MELD score, and use of fluoroquinolones. The stepwise variable selection process identified 2 variables independently associated with 30-day readmission. A greater hazard of readmission was found for patients with increased Charlson Comorbidity Index (HR 1.12; 95% CI: 1.03–1.23) and higher MELD score (HR 1.04; 95% CI: 1.01–1.07).

**Table 3.**
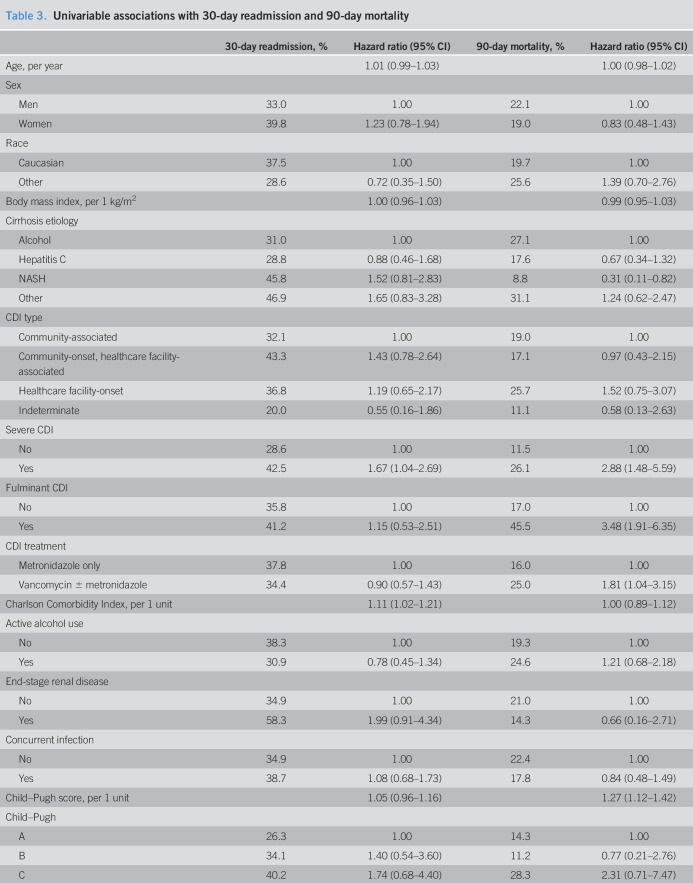

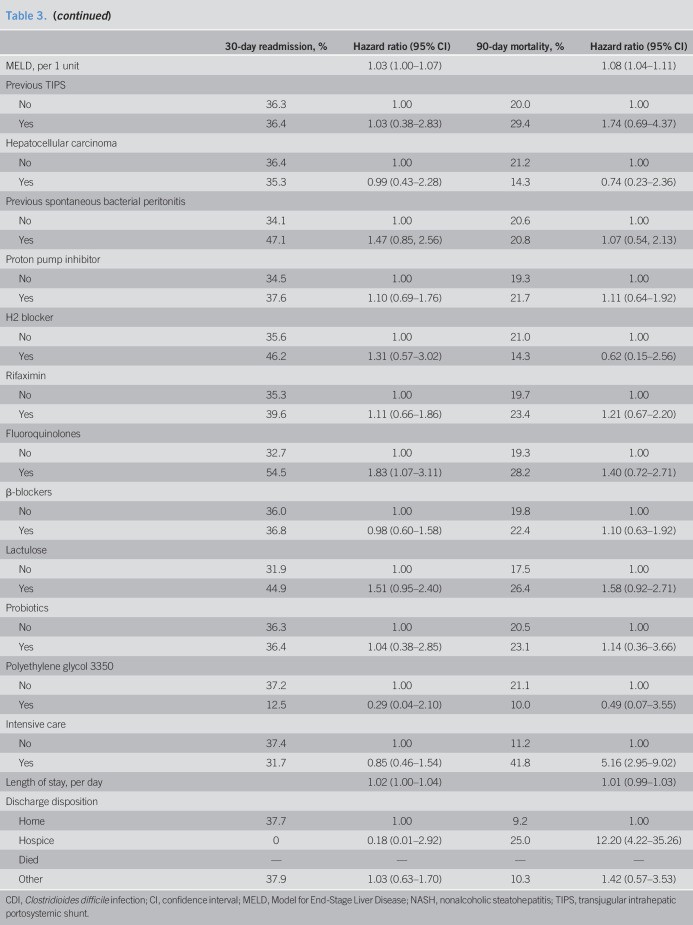
Univariable associations with 30-day readmission and 90-day mortality

### Ninety-day mortality

Of the 257 patients, 53 died within 90 days (28 during the index admission and 25 after discharge), leading to an estimated mortality rate of 22.8% (Figure 3b). In those without R-CDI, 90-day mortality was 20.8%. Death was associated with nonalcoholic steatohepatitis etiology of cirrhosis, severe CDI, fulminant CDI, treatment with vancomycin, increased Child–Pugh score, increased MELD score, and intensive care (Table 3). In multivariable analysis, mortality was associated with cirrhosis etiology (alcohol HR 2.67; 95% CI: 0.99–7.22; hepatitis C HR 1.34; 95% CI: 0.48–3.72; other etiology HR 3.31; 95% CI: 1.19–9.25; reference: nonalcoholic steatohepatitis), increased Child–Pugh score (HR 1.24; 95% CI: 1.11–1.39) and intensive care (HR 5.47; 95% CI: 3.05–9.84).

## DISCUSSION

This large cohort study of more than 200 patients is the first, to our knowledge, to examine the incidence, risk factors, and outcomes of CDI recurrence in patients with cirrhosis. The incidence of R-CDI in this study was 11.9%, which is on the low end of rates reported in other populations. In a landmark randomized trial of CDI treatment, R-CDI occurred in 25% of patients treated with vancomycin and in 15% of those treated with fidaxomicin (20). Another large, population-based study found an R-CDI incidence of 14% (21). A systematic review of 33 studies found a median recurrence rate of 22% (range 10%–50%) across different populations (22). Two large retrospective studies also found R-CDI rates of 10% (23,24). Differences between studies could be due to differences in R-CDI definitions (e.g., different time frames for recurrence and requirements for repeat laboratory testing). In this study, we used the guideline-based definition of R-CDI, which might favor a lower estimate compared with other, more liberal R-CDI definitions (14). Other reasons for higher rates seen in other studies include the potential for detection bias: patients followed up prospectively in a clinical trial protocol might be more likely to report and seek treatment for recurrent diarrhea. Notably, although other chronic conditions (e.g., chronic kidney disease [CKD]) are known risk factors of R-CDI (22,25), cirrhosis has not been associated with an increased risk of R-CDI (24,26).

We found that R-CDI is independently associated with an increased comorbidity burden. This observation confirms similar findings in multiple other studies in different populations (22,23,27). In addition to the overall comorbidity index, R-CDI has been associated with several individual comorbidities, including diabetes and most prominently, CKD (24,25,28). Beyond CKD, the risk of R-CDI might be even greater in those with end-stage renal disease (25). Although we did not find a significant association with end-stage renal disease, the rate of R-CDI was numerically greater in this group (29.3% vs 11.9%; *P* = 0.11); the lack of a significant association might be related in part to the limited number of patients with end-stage renal disease. Increased age has been found to be a risk factor of R-CDI in multiple studies (22). We found an association with age in univariable analysis but not in the multivariable analysis. This finding is likely due to the inclusion of age as a component of the comorbidity index. Last, in addition to its association with R-CDI, an increased Charlson Comorbidity Index has also been associated with other poor CDI outcomes, including disease severity (29) and in our study, hospital readmission.

The other factor independently associated with R-CDI was lactulose use. This finding was unexpected and contradicts other data. Previous *in vitro* work showed that lactulose-induced stool acidification suppresses fecal anaerobes, in particular *C. difficile* (30). Furthermore, in a case–control study by Agarwalla et al. (31), lactulose use was associated with a reduced risk of initial CDI in hospitalized patients with decompensated cirrhosis. This discrepancy might be explained by important differences between this study and that by Agarwalla et al.. First, the study by Agarwalla et al. examined initial CDI, as opposed to R-CDI in our study. Different risk factors for initial vs R-CDI have been shown in other populations (32). Second, in the study by Agarwalla et al., patients with CDI were less likely to have hepatic encephalopathy and rifaximin use compared with patients with cirrhosis without CDI. Although the protective effect of lactulose on CDI was maintained after adjustment for rifaximin use, the authors were unable to adjust for hepatic encephalopathy because of the high correlation with rifaximin use. By contrast, we found a trend toward increased R-CDI in those receiving rifaximin (20.2% vs 9.3%; *P* = 0.06), consistent with findings from other studies demonstrating increasing rifaximin-resistant strains of CDI (33). Finally, lactulose use in our study might simply be a surrogate marker of liver disease severity; Lactulose use was associated with a greater Child–Pugh score, a higher MELD score, and more frequent cirrhosis complications such as hepatic encephalopathy, ascites, varices, and spontaneous bacterial peritonitis.

Antibiotics and proton pump inhibitors are well-known risk factors of initial CDI and R-CDI, but neither was associated with R-CDI in our study. This finding might be due to widespread use of these medications in this population, which might have predisposed the patients in this cohort to develop CDI in the first place. Notably, the proportion of patients receiving either proton pump inhibitors or antibiotics did not decrease on hospital discharge compared with admission. Improving medication stewardship should remain a primary goal in the effort to reduce R-CDI in cirrhosis.

In addition to R-CDI, we examined other patient outcomes including hospital readmission and mortality. Our 90-day mortality (22.8%) is lower than the mortality in another single-center study (44% at 30 days) (34) but greater than the 13.8% in-hospital mortality seen in national inpatient data (13). Risk factors for mortality in our study included increased Child–Pugh score and intensive care, consistent with previous work (34). Thirty-day readmissions occurred in 37% and were associated with increased MELD score and comorbidity burden. This rate is slightly higher than the pooled readmission rate of 26% across different studies of patients with cirrhosis (35), consistent with findings that patients with cirrhosis and CDI have worse outcomes compared with those of patients with cirrhosis without CDI (after controlling for age, comorbidities, cirrhosis complications, and other infections) (13). Notably, MELD score is a well-established risk factor of readmission in patients with cirrhosis (35).

We acknowledge several potential limitations to this study. Its retrospective design makes it vulnerable to information biases. We minimized any potential for such error through rigorous standardized data abstraction to ensure consistency across the study subjects. Furthermore, any misclassification is likely to be nondifferential, which would bias findings toward the null. Indiana University Hospital is a tertiary referral center, and the study cohort might not reflect the larger population of patients with cirrhosis and CDI. In addition, R-CDI could be misclassified if patients do not receive care for the recurrence at our center. However, most patients with end-stage liver disease obtain their care at referral centers, and they are more likely to return for treatment of recurrence if the initial CDI was treated at our center. Finally, nearly half of the cohort was treated with metronidazole monotherapy, which is no longer recommended for treatment of initial CDI in recently published practice guidelines (14). However, R-CDI rates in our study were no different in those who received vancomycin vs metronidazole alone, suggesting that our observed risk factors for R-CDI are likely to be relevant in the new, current treatment paradigm. Our study benefits from a large sample size compared with previous studies, with detailed phenotyping of both liver disease and CDI characteristics. However, the relatively low rate of R-CDI likely limits our power to identify specific risk factors.

In conclusions, we found a rate of R-CDI in patients with cirrhosis of 11.9%, exacerbated by a high comorbidity burden and the use of lactulose. Patients with these risk factors might benefit from interventions to prevent recurrent disease and improve patient outcomes. Careful attention to medication usage should remain a focus of these efforts.

## CONFLICTS OF INTEREST

**Guarantor of the article:** Eric S. Orman, MD.

**Specific author contributions:** P.P.: planning and conducting the study, collecting data, and drafting the manuscript, R.D.P., D.G.: collecting data and drafting the manuscript, H.X.: interpreting data and drafting the manuscript, K.B.-B.: collecting data and editing the manuscript, N.C., M.F.: interpreting data and editing the manuscript, E.S.O.: planning and conducting the study and drafting and reviewing the manuscript. Every author has approved the final draft to be submitted.

**Financial support:** This work was supported, in part, by the National Institute of Diabetes and Digestive and Kidney Diseases of the National Institutes of Health under award number K23DK109202. The sponsors played no role in the study design, collection, analysis, or interpretation of the data, or in the writing of the report. The work was independent of the funding.

**Potential competing interests:** None to report.

**Study Highlights**

**WHAT IS KNOWN**

✓ CDI is common in patients with cirrhosis and is associated with poor outcomes.
✓ Patients with cirrhosis have high rates of hospitalization and increased exposure to antibiotics and proton pump inhibitors, which are known risk factors of CDI.

**WHAT IS NEW HERE**

✓ The incidence of R-CDI in patients with cirrhosis is similar to the incidence in other populations.
✓ Risk factors of R-CDI in patients with cirrhosis include increased comorbidity burden and the use of lactulose.

**TRANSLATIONAL IMPACT**

✓ High-risk patients with cirrhosis and CDI might benefit from interventions to prevent R-CDI.
